# OGT Protein Interaction Network (OGT-PIN): A Curated Database of Experimentally Identified Interaction Proteins of OGT

**DOI:** 10.3390/ijms22179620

**Published:** 2021-09-06

**Authors:** Junfeng Ma, Chunyan Hou, Yaoxiang Li, Shufu Chen, Ci Wu

**Affiliations:** 1 Lombardi Comprehensive Cancer Center, Department of Oncology, Georgetown University Medical Center, Washington, DC 20057, USA; lzyacht@gmail.com (Y.L.); cw1056@georgetown.edu (C.W.); 2 Dalian Institute of Chemical Physics, Chinese Academy of Sciences, Dalian 116023, China; scenery119@163.com; 3 School of Engineering, Pennsylvania State University Behrend, Erie, PA 16563, USA; ringosimonchen0820@gmail.com

**Keywords:** database, O-GlcNAc, OGT, OGT interactome, protein–protein interactions

## Abstract

Interactions between proteins are essential to any cellular process and constitute the basis for molecular networks that determine the functional state of a cell. With the technical advances in recent years, an astonishingly high number of protein–protein interactions has been revealed. However, the interactome of O-linked N-acetylglucosamine transferase (OGT), the sole enzyme adding the O-linked β-N-acetylglucosamine (O-GlcNAc) onto its target proteins, has been largely undefined. To that end, we collated OGT interaction proteins experimentally identified in the past several decades. Rigorous curation of datasets from public repositories and O-GlcNAc-focused publications led to the identification of up to 929 high-stringency OGT interactors from multiple species studied (including *Homo sapiens*, *Mus musculus*, *Rattus norvegicus*, *Drosophila melanogaster*, *Arabidopsis thaliana*, and others). Among them, 784 human proteins were found to be interactors of human OGT. Moreover, these proteins spanned a very diverse range of functional classes (e.g., DNA repair, RNA metabolism, translational regulation, and cell cycle), with significant enrichment in regulating transcription and (co)translation. Our dataset demonstrates that OGT is likely a hub protein in cells. A webserver OGT-Protein Interaction Network (OGT-PIN) has also been created, which is freely accessible.

## 1. Introduction

Since its discovery in the early 1980s [1,2], O-linked β-N-acetylglucosamine (O-GlcNAc) has been gradually established as an essential post-translational modification of proteins (i.e., O-GlcNAcylation). Distinct from other types of glycosylation, O-GlcNAc is a unique intracellular monosaccharide modification on serine/threonine residues of nuclear/cytoplasmic and mitochondrial proteins [3,4]. It has been found that O-GlcNAcylation occurs on >5000 proteins spanning a range of species, as can be seen from the rigorously curated database O-GlcNAcAtlas [5]. Numerous evidence has demonstrated that the molecular diversity of O-GlcNAcylated proteins has a fundamental importance in many biological processes in physiology and pathology [6,7,8,9,10,11]. Targeting protein O-GlcNAcylation holds great promise for the development of therapeutic targets and biomarkers [12]. Interestingly, despite the substrate diversity, O-GlcNAcylation is catalyzed by only a pair of enzymes: O-GlcNAc transferase (OGT) adds O-GlcNAc onto proteins while O-GlcNAcase (OGA) removes it from proteins [13,14]. Recent years have witnessed great progress towards the understanding of OGT, with deep structural insights obtained and multiple functions revealed [15,16,17,18,19,20]. However, the modes and mechanisms of how OGT works (e.g., interacting with other proteins) have been intriguing and largely unknown.

Mapping protein–protein interactions (PPIs) is instrumental for understanding both the functions of individual proteins and the functional organization of the cell as a whole [21,22,23]. Given the huge importance, a wide array of methods has been developed to probe PPIs in vitro, ex vivo, or in vivo, including yeast two-hybrid (Y2H), protein microarrays, co-immunoprecipitation, affinity chromatography, tandem affinity purification, fluorescence resonance energy transfers (FRET)-related techniques, X-ray crystallography, NMR spectroscopy, and mass spectrometry-based approaches [24,25,26]. Of note, some high throughput methods, especially those coupled with tandem mass spectrometry (MS/MS) (e.g., affinity purification MS (AP-MS), immunoprecipitation-MS (IP-MS), cross-linking MS (XL-MS), proximity labeling MS (PL-MS), and protein correlation profiling MS (PCP-MS)), have enabled the global characterization of PPIs (i.e., interactomics) [27,28,29]. As a critical protein involved in many biological processes, OGT, together with its binding partners, has been unsurprisingly identified from numerous studies. Of note, such methods have also been specifically tailored for the characterization of OGT-interacting proteins recently [30,31,32,33,34,35].

To accommodate the exponentially increased datasets of PPIs, a plethora of comprehensive and specific databases (e.g., BioGRID [36], APID [37], IntAct [38], HuRI [39], HIPPIE [40], HRPD [41], STRING [42], PlaPPISite [43]) have been constructed. These public repertories categorize hundreds and thousands of PPIs from many species. However, after surveying through the >2000 O-GlcNAc-focused studies published previously [5], we found that only a limited number of OGT-interacting proteins had been described in these databases. Furthermore, information of OGT-interactors is sparsely distributed in multiple repositories, with differential stringency applied. To that end, we compiled a rigorously curated and comprehensive database specifically for interaction proteins of OGT and its orthologues identified (e.g., SXC in *Drosophila melanogaster*, SEC in plants, and OGT-1 in *Caenorhabditis elegans*), with the goal to provide researchers a rigorously curated but in-depth database for high-stringency OGT interactors. A webserver OGT-PIN (https://oglcnac.org/ogt-pin/) was also constructed, with the hope to better serve investigators in the glycoscience community and beyond.

## 2. Results and Discussion

### 2.1. A Comprehensive and High-Stringency Database of OGT-Interacting Proteins

With the technical advances, a number of comprehensive and specific protein interaction databases have been constructed in recent years. Although a joint International Molecular Exchange (IMEx) consortium curation manual (http://www.imexconsortium.org/curation/; version 1 May 2015) was proposed [44], each database appears to have its focus and covers only a portion of protein entries [45]. Given the quickly evolving techniques (especially those for the high throughput analytical characterization), astronomic amount of data is being produced in an unprecedented manner, rendering construction of a unified database for all protein interactions a daunting task.

**Figure 1:**
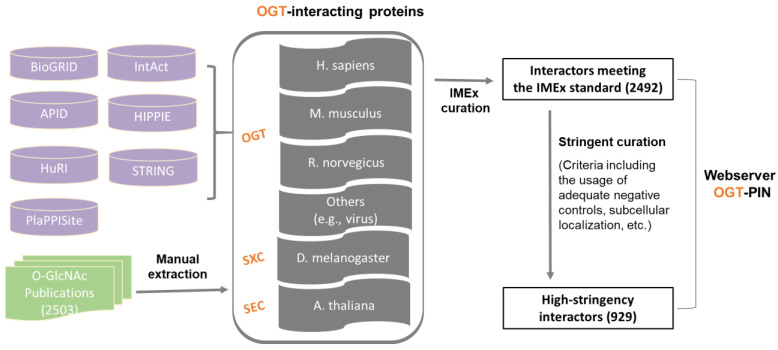
Assembly of the experimentally identified OGT-interacting protein database. In total, information of OGT interactors was extracted from seven public repositories (i.e., BioGRID, APID, IntAct, HuRI, HIPPIE, STRING, and PlaPPISite) and 2503 O-GlcNAc-focused publications. OGT interactors were first curated by following the single joint International Molecular Exchange (IMEx) consortium curation manual and then curated with more stringent criteria (as described in detail in ‘Methods’). Information of both interactors meeting the IMEx standard and interactors with high-stringency was included in the webserver OGT-PIN.

In this study, we aimed to create a compendium of interacting proteins of OGT and orthologues identified in the past several decades. The OGT interactome database was built using a combination of data retrieved from public repositories and manual extraction and curation from O-GlcNAc-focused studies (both small-scale and large-scale) published previously (as shown in Figure 1). Specifically, we retrieved OGT-interacting proteins from comprehensive databases and specific ones (including BioGRID, APID, IntAct, HuRI, HIPPIE, STRING, and PlaPPISite) that contain hundreds and/or thousands of protein pairs. Besides OGT, several relatively well-characterized OGT orthologues (including SXC in *Drosophila melanogaster*, SEC in plants, and OGT-1 in *Caenorhabditis elegans*) were used for searching. Each database was found to contain only a small portion of OGT interaction proteins (not shown). Moreover, there appeared to be relatively small overlaps between databases. To make a complete compendium, manual extraction and curation (by following the single joint IMEx curation manual) were performed to 2503 O-GlcNAc-centered studies from 1984 to 30 December 2020. The combined dataset gave rise to a total of 2492 experimentally identified interaction proteins (Supplementary Table S1).

Although the IMEx consortium curation manual provides a basic and general guideline for data curation, we found substantial stringency differences between different datasets. More importantly, there was a need to define high-stringency OGT interactors with more stringent rules. To that end, further curation was performed to the resulting list of proteins meeting the IMEx curation manual. First of all, we noticed that a number of entries from public repertories did not have original sources (e.g., PubMed numbers). Such items were excluded, due to the inability to evaluate those interactors. Moreover, special attention was paid to entries curated from high throughput studies. Although entries from original experiments without adequate negative controls were labeled as ‘Caution’ per the recommendation of IMEx curation manual, such items were excluded from the high-stringency list. In addition, subcellular localization was applied to all entries. Considering OGT interaction has been almost exclusively observed in nuclear/cytosol/mitochondria so far, interactors localized to other organelles (e.g., extracellular space, plasma membrane, Endoplasmic reticulum, and Golgi apparatus) were excluded. This two-step curation strategy yielded a list of 929 high-stringency interaction proteins of OGT and orthologues (from a total of 221 publications) (Supplementary Table S1).

**Figure 2:**
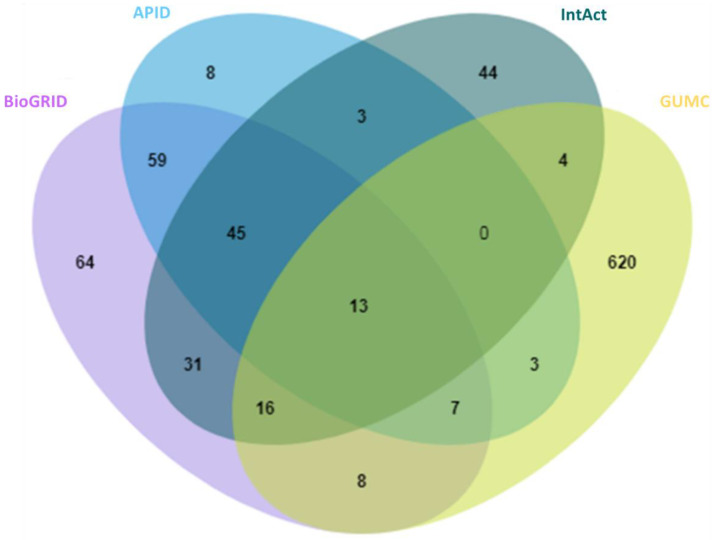
Overlap of 929 high-stringency OGT-interacting proteins from different sources, including BioGRID, APID, IntAct, and GUMC (denoting OGT-interacting proteins additionally extracted and curated by the team at the Georgetown University Medical Center). Curated OGT-interacting proteins obtained from HIPPIE, HuRI, and PlaPPISite were not included for this comparison, due to the presence of only a few additional proteins.

With anticipation, a small overlap between public repertories was observed for the high-stringency OGT interactors (Figure 2). In addition, 620 proteins were additionally curated from O-GlcNAc-centered studies (extracted from 63 publications). One reason for missing so many OGT interaction proteins by public repositories is probably due to the focus of different curators—they may mostly have focused on PPI studies in general while we paid special attention to OGT-involving PPIs from a relatively smaller cohort of publications. These results further demonstrate that there is still a need to construct in-depth protein interaction databases by manual extraction and curation for specific molecules of special interest (as also exemplified for interactomes of phosphatase [46] and heparin/heparan sulfate [47,48]). Last but not least, we believe that data quality of a database is of ultimate importance. Given the false positives reported in the literature (e.g., due to the lack of adequate controls especially in high-throughput proteomic studies), clearly there is a need to treat such entries seriously by curators of every database—to minimize and avoid misleading to users. Adoption of high-stringency and unified curation criteria renders the generation of a database of high confident protein interactors for OGT. 

**Figure 3:**
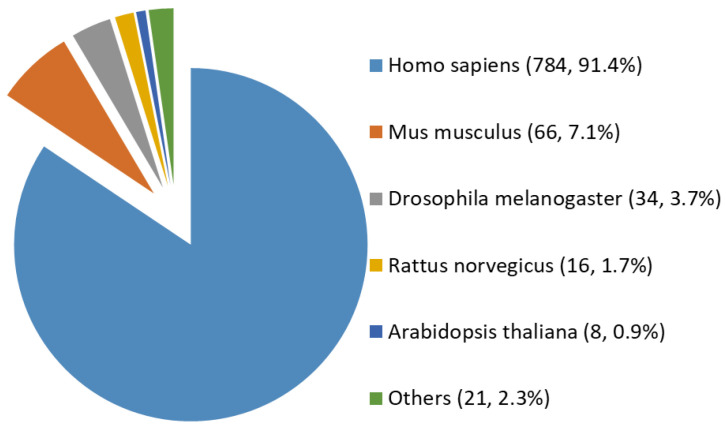
Species distribution of 929 high-stringency interacting proteins of OGT and orthologues. Other interactor proteins include seven from Influenza A virus strains, three from *Bacillus anthracis*, three from *Campylobacter jejuni subsp. jejuni serotype O:2* (strain ATCC 700819/NCTC 11168), two from *Caenorhabditis elegans*, two from Severe acute respiratory syndrome coronavirus 2 (2019-nCoV) (SARS-CoV-2), one from Human cytomegalovirus (strain Merlin) (HHV-5) (Human herpesvirus 5), one from *Oryctolagus cuniculus*, one from *Francisella tularensis subsp. tularensis* (strain SCHU S4/Schu 4), and one from *Sus scrofa* (pig).

Among all the 929 high-stringency interaction proteins, over half were identified by AP-MS, the most popular tool for protein interatomic studies nowadays. The 929 proteins were found distributed in multiple species investigated, including *Homo sapiens*, *Mus musculus*, *Rattus norvegicus*, *Drosophila melanogaster*, *Arabidopsis thaliana*, and a few others (e.g., *Caenorhabditis elegans*, Influenza A virus strains, and SARS-CoV-2). Among them, ~84% (784 human proteins) were interaction partners of human OGT (Figure 3). Moreover, the majority (87%) of OGT interactors were found once, while ~13% were identified by two or more independent studies (Supplementary Figure S1).

**Figure 4:**
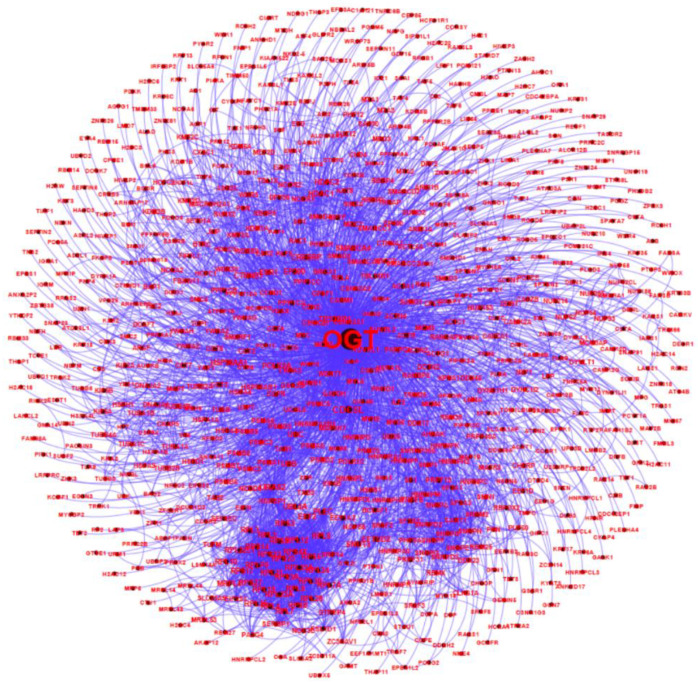
A human OGT interaction network consisting of 782 nodes and 4224 edges (protein interactions). Some nodes are shown in a bigger size due to their higher numbers of associations with other proteins.

All of the 784 human proteins interacting with human OGT were used to construct a human OGT interactome network. Because the partners of the OGT interactors may interact with each other in vivo, such interactions were extracted by querying STRING. Gephi [49] was used to visualize the OGT network consisting of 782 nodes and 4224 edges (protein interactions) (Figure 4). The highly connected nodes tend to make clusters and hubs in a dense network (the average number of interactions for a node is up to 10.8). A word cloud representation of all interactors of human OGT is shown in Supplementary Figure S2. A list of the top 18 frequently identified interaction proteins is shown in Table 1, including a number of relatively well-characterized interactors by biochemical approaches (e.g., HCFC1, OGT, OGA, TET1, TET2, and TAB1).

### 2.2. OGT-Interacting Proteins and OGT Substrate Proteins

**Figure 5:**
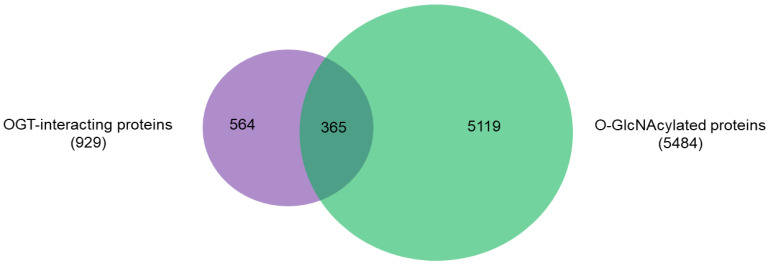
Overlap between 929 high-stringency OGT-interacting proteins in OGT-PIN and O-GlcNAcylated proteins in O-GlcNAcAtlas (Version_01.08).

An intriguing aspect to understand OGT functions is to distinguish its interacting proteins from its substrate proteins. To that end, we compared the 929 high-stringency interaction proteins in our OGT interactome database OGT-PIN with O-GlcNAcAtlas (https://oglcnac.org/; version_01.08), a comprehensive and highly curated database for O-GlcNAc proteins and sites [5]. Very strikingly, it appears that only a small percentage (~39%) of OGT-interacting proteins are OGT substrates (Figure 5), supporting the notion that OGT interactors are not necessarily OGT substrates. Indeed, some of the OGT interactors are also good OGT substrates (e.g., HCFC1, OGT, OGA, TET1, TET2, and TAB1). With the further technical advances in O-GlcNAc site mapping techniques, more OGT-interacting proteins might be found O-GlcNAcylated. But it appears that others (including some highly frequently identified OGT interactors including BAP1, WDR5, FBXW11, and RBBP5 shown in Table 1) are not O-GlcNAcylated. Clearly, the functional roles of such proteins in OGT biology are worthy for further exploration.

Recent studies have revealed that OGT is in fact a multi-faceted protein, besides serving as the sole enzyme catalyzing O-GlcNAcylation on thousands of proteins. So far at least four other functions of OGT have been discovered: (1) catalyzes site-specific proteolysis of a transcriptional coactivator HCFC1 [50]; (2) transfers GlcNAc to cysteines (i.e., S-GlcNAc) of cellular proteins [51,52]; (3) use UDP-glucose to install O-linked glucose (O-Glc) onto proteins [53,54]; and (4) catalyzes aspartate to isoaspartate isomerization [55]. The list of high-stringency interaction proteins of OGT will likely provide clues to further understand these non-canonical functions and other functions of OGT yet to be elucidated. 

### 2.3. Functional Diversity of OGT-Interacting Proteins

**Figure 6:**
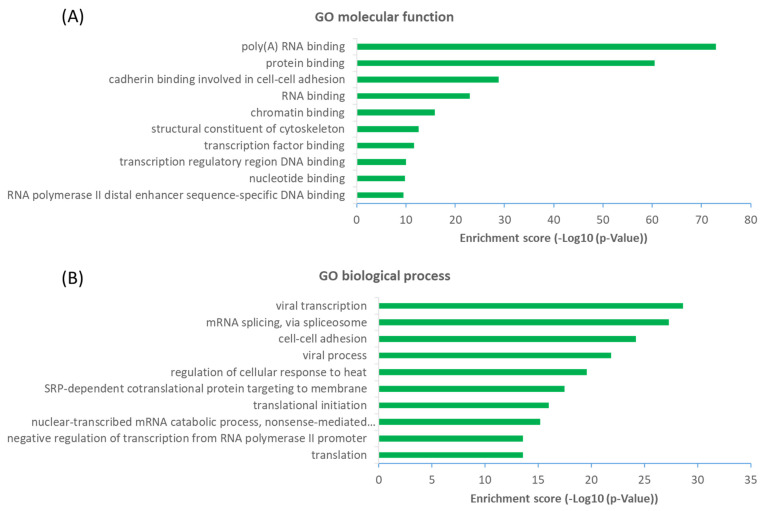
Functional landscape of 784 high-stringency OGT-interacting proteins in human, according to their GO molecular functions (**A**) and biological processes (**B**). Only the top ten items with the highest enrichment scores are shown.

The observation that a large proportion (~61%) of interactors are not OGT substrate proteins is very intriguing. Next, we investigated the potential functions of human OGT-interacting proteins. Remarkably, gene ontology (GO) analysis revealed a highly significant enrichment of proteins with the molecular function terms ‘Poly(A) RNA-binding’ and ‘RNA-binding’ (Figure 6A). Concomitantly, ‘transcription’ and ‘(co)translation’ seemed to be the highly enriched biological processes (Figure 6B). 

**Figure 7:**
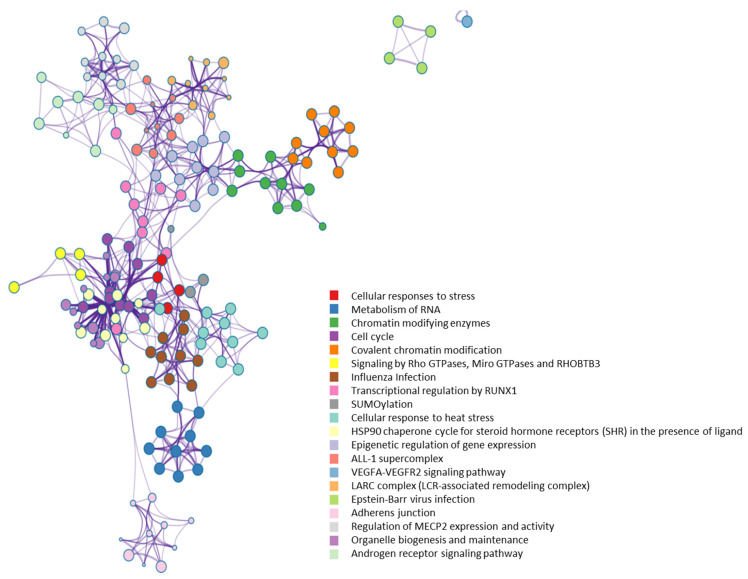
Highly clustered modules of OGT-interacting network. Each term is represented by a circle node, where its size is proportional to the number of input genes that fall into that term, and its color represents its cluster identity (i.e., nodes of the same color belong to the same cluster).

From a molecular network perspective, highly clustered modules of OGT interactors were predominantly involved in RNA metabolism, RNA splicing, ribonucleoprotein complexes, chromatin modifications, and others (Figure 7). Since RNA binding proteins play a critical role in controlling various aspects of transcript and translation (including mRNA stability and translation efficiency), the ubiquitous distribution of OGT interactors on transcriptional/translational machinery and other relevant complexes might be a key contributor to the well-documented transcriptional/translational regulation by protein O-GlcNAcylation [10,56,57,58]. Of note, it appears that the interaction partners of OGT were also strongly enriched in proteins involved in cellular responses to stress (Figure 7), in which O-GlcNAcylation has been found to play an important role as well [59].

### 2.4. OGT as a likely Hub Protein in Cellular Interaction Network

Such a high number of OGT interactors is somewhat unexpected since OGT has not been considered as a hub protein yet [60]. Apparently, OGT has a comparable or even higher number of interacting proteins than many of the ~300 hub proteins (each has several hundred interactors) [60]. Furthermore, OGT is functionally essential since the knockout of OGT is embryonically lethal in a number of organisms [61], fitting well with the classic centrality-lethality rule [62]. Therefore, OGT is likely a hub protein in the cellular network. 

The high number of OGT interactors might be closely related to its unique properties. Catalytically, OGT is the sole enzyme that can add O-GlcNAc to thousands of substrate proteins in nuclear, cytosol, and mitochondria. This is distinct from many other enzymes (e.g., glycosyltransferases, kinases, phosphatases, ubiquitin ligases, sirtuins) which often have multiple family members to concertedly modify hundreds or thousands of proteins. It is largely unclear why and how nature chooses OGT to fulfill its duties in such a ubiquitous manner. To achieve that, one possibility is that some OGT interactors serve as scaffold, anchoring, or adaptor proteins that contribute to recruiting active OGT molecules into cellular complexes or by placing OGT close to their substrates, as they do for other post-translational modifications (e.g., phosphorylation) [63,64]. Indeed, among the OGT interactors, quite a few are well-known scaffold proteins (e.g., several 14-3-3 family proteins including YWHAE, YWHAG, YWHAH, and YWHAZ), anchoring proteins (e.g., AKAP2, AKPA12), and adaptor proteins (e.g., importin α). Interestingly, besides binding to OGT, importin α and 14-3-3 proteins also have demonstrated evolutionarily conserved O-GlcNAc binding properties that can directly and selectively recognize/read O-GlcNAc moieties on proteins [65,66,67]. 

A structural perspective may help partially explain why OGT has so many interactors. OGT has mainly two regions: An N-terminal region consisting of a series of tetratricopeptide repeat (TPR) units (containing 34 amino acids in each) and a multi-domain catalytic C-terminal region. The TPR domains of proteins generally mediate protein–protein interactions and the assembly of multiprotein complexes [68,69]. Although the TPR structural motif is present in many proteins (predicted to be up to 260) [68], human OGT contains a super-helical TPR domain consisting of a very high number of TRP units (13.5). Moreover, the TPR domain appears to be the location where the OGT homotrimer/heterotrimer forms. Crystal structure studies of OGT reveal that TPR superhelix consists of two layers of helices, an inner concave face formed by helix-A and an outer convex face formed by helix-B [15,16,17,18]. The resulting wide binding surface is likely to present several overlapping binding pockets that can hold multiple substrates/interactors. It appears that the conserved asparagine and aspartate ladders regulate the binding of interacting proteins by forming bidentate hydrogen bonds with the peptide backbone [70,71]. In addition, the C-terminal region (e.g., the intervening-D domain and the C-terminal putative phosphatidylinositol-3,4,5-trisphosphate-binding domain) might also be involved in the recognition and binding of versatile proteins [19,20,72]. 

Despite the great progress especially in the past decade, further studies (e.g., resolving structures of OGT and protein interactors) should promote understanding of detailed interaction mechanisms between OGT and its diverse interacting proteins. 

### 2.5. Webserver OGT-PIN

**Figure 8:**
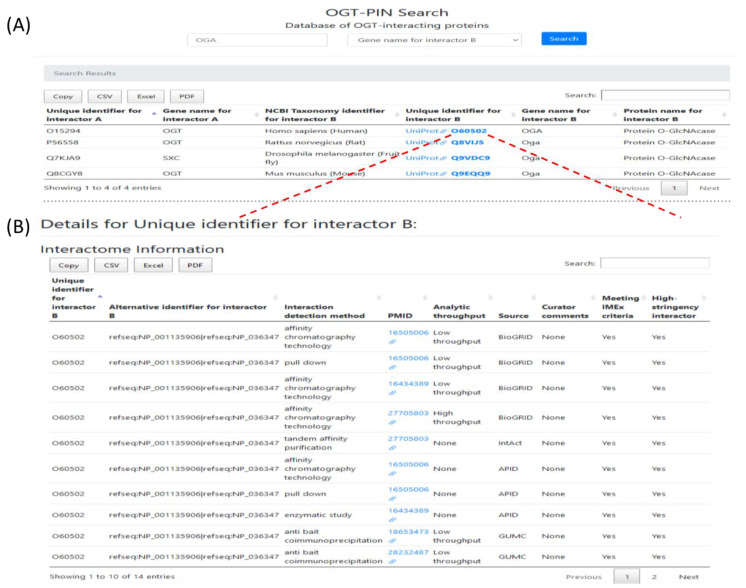
A snapshot for searching the webserver OGT Protein Interaction Network (OGT-PIN), with ‘Protein O-GlcNAcase (OGA)’ as an example. The top panel (**A**) are the tabular results for all the four matched entries identified from different species, and the bottom panel (**B**) shows detailed information of 14 entries matched with human OGA, with links to the original studies in PubMed. Among the 14 entries meeting IMEx criteria, one entry was excluded from the high-stringency list.

To facilitate the use of this resource, a web interface OGT-PIN was created and made freely accessible at https://oglcnac.org/ogt-pin/. Users can browse and search efficiently for OGT-interacting proteins of interest. OGT-PIN can be searched using UniProt accession for interactor A, gene symbol for interactor A, UniProt accession for interactor B, gene symbol for interactor B, and species of interacting proteins. The results can be filtered further, according to specific needs. The search output includes the basic annotations for all the matched entries (as exemplified in Figure 8A). The accession number of each entry is linked to the detailed information for specific proteins (including gene/protein name of the interactor protein, interaction detection method, analytical throughput, PubMed ID of the original article, and curators’ comments; as exemplified in Figure 8B). Currently, OGT-PIN supports several functions including data searching, browsing, and retrieving. In addition, search results can be directly downloaded and saved from the OGT-PIN webpage. Of special note, both entries meeting with the IMEx curation manual and entries meeting our high-stringency criteria have been kept in the database. Users are encouraged to explore the details of each entry via the linked original articles and use entries that meet high-stringency criteria as high confident OGT interactors.

## 3. Methods

### 3.1. OGT-Interactome Database

The schematic workflow for assembly of the OGT-interacting protein database is presented in Figure 1.

Specifically, data from public repertories were obtained from seven public databases (namely BioGRID, APID, IntAct, HuRI, HIPPIE, STRING, and PlaPPISite) by searching with the accession number or gene symbol of OGT and its orthologues (including SXC, OGT-1, and SEC) (accessed in January 2021). Another set of data was obtained by manual extraction of relevant information from O-GlcNAc-focused publications. In total, by using a similar approach described previously [5], 2503 publications (including 2236 from 1984 to 2019 and 267 in 2020) were obtained and used for manual extraction and curation. A two-step curation strategy was performed for all entries obtained. First, OGT interactors were curated by following the single joint International Molecular Exchange (IMEx) consortium curation manual (http://www.imexconsortium.org/curation/; version 1 May 2015) [44]. Information of OGT-interacting proteins in each publication was recorded, with the resulting dataset termed as GUMC (i.e., by the Georgetown University Medical Center curation team). Related information (including species, analytical methods used, and the corresponding PubMed ID) of PPIs was also extracted. To unify different data formats, all experimentally identified proteins were mapped to the UniProtKB database [73] for protein entries and gene symbols. To maintain integrity, entries that could not be matched with UniProt accession numbers were also kept in the dataset with all information available. According to the IMEx consortium curation manual, curators’ comments (e.g., ‘Caution’) were given to certain entries that seem to lack validity according to the methods described in the original studies. Once available, quantitative scores in the original studies were presented in the curators’ comments as well. 

To create a list of high-stringency interactors of OGT and its orthologues, further curation was conducted to the list of proteins meeting IMEx rules. Several criteria were applied: (1) entries from public repertories that do not have original sources (e.g., PubMed numbers; except those in preprints) were excluded, due to the inability to evaluate such interactors; (2) special attention was paid to entries from high throughput proteomic studies—entries resulted from original experiments without adequate negative controls were excluded (no matter whether they have been curated by public repertories or the GUMC team according to the IMEx manual); (3) entries that could not be curated (e.g., due to the failure to be assigned to a specific gene/accession) were excluded, with the goal to further minimize potential ambiguity; and (4) subcellular localization was applied to all entries—since OGT interaction has been almost exclusively observed in nuclear/cytosol/mitochondria, interactors localized to other organelles (e.g., extracellular space, plasma membrane, endoplasmic reticulum, and Golgi apparatus) were excluded. To respect the relevant work from original authors, to follow the guidelines from IMEx curation manual, and also to maintain scientific soundness for high-stringency requirements, both entries meeting with the IMEx curation manual and entries meeting our high-stringency criteria were kept and clearly labeled in the final dataset (Supplementary Table S1). But only proteins meeting high-stringency criteria were regarded as high confident interactors for OGT.

### 3.2. Bioinformatic Analysis

Gene Ontology (GO) analysis [74] and Bologna Unified Subcellular Component Annotator (BUSCA) [75] were used to evaluate the subcellular localization of proteins. Venn diagrams for protein overlapping were created by using EVenn [76]. STRING [42] and Gephi [49] were used to construct an interaction network consisting of human OGT-interacting proteins. GO analysis was performed to identify the biological processes and molecular functions of enriched human interaction proteins of human OGT. DAVID [77] and Metascape [78] were used for the extraction of enrichment clusters. Terms with a similarity score >0.3 were linked by an edge (the thickness of the edge represents the similarity score). The network was visualized by Cytoscape (v3.1.2) with a “force-directed” layout and with edge bundled for clarity. One term from each cluster was selected to have its term description shown as labeled. 

### 3.3. Construction of Webserver OGT-PIN

A user-friendly web server OGT-PIN was created by using a similar approach as for O-GlcNAcAtlas. Specifically, the web graphical user interface was created with HTML, CSS, and Bootstrap. Python programming language (version 3.8.1) coupled with the MySQL database was used to develop the backend server. For simplicity, OGT and its orthologues were considered as interactor A and the corresponding protein partners as interactor B (regardless of whether the proteins were used as bait or prey in initial studies). The resulting dataset, with each entry given a unique identification number, was organized in the MySQL database. For each interactor, detailed information (including accession number and gene/protein name of OGT and its interactors, interaction detection method, analytical throughput, PubMed ID of the original article, and curators’ comments) was provided. Each PubMed ID was linked to the original study that identified the interacting protein. To facilitate data retrieval, OGT-PIN was made freely accessible at https://oglcnac.org/ogt-pin/.

As an example, OGA, a relatively well-characterized interactor of OGT, was searched against the OGT-PIN. A snapshot of the results is shown in Figure 8. The top panel is the tabular results for all the four matched entries identified from different species (Figure 8A), while the bottom panel shows detailed information of 14 entries matched with human OGA (Figure 8B).

## 4. Conclusions

The quickly evolving analytical technologies have yielded an enormous amount of protein–protein interaction data, especially in recent years. By combining the datasets from major public repertories and manual extraction and curation of O-GlcNAc-focused studies (both small-scale and large-scale ones), we created a rigorously curated and comprehensive database of OGT-interacting proteins experimentally identified in the past several decades. 

Different from public repertories, a two-step curation strategy (by observing both IMEx curation guidelines and our stringent criteria of protein interactors specifically for OGT) was adopted, yielding a list of 929 high-stringency interaction proteins of OGT and orthologues (including 784 proteins interacting with human OGT). Interestingly, only a small percentage (~39%) of OGT-interacting proteins have been identified as OGT substrates. Considering the versatile functions of the diverse interactors, OGT is likely another hub protein in a highly connected cellular network. 

We anticipate this reference resource can provide insights into our understanding of OGT biology and protein O-GlcNAcylation. It may also serve as a useful starting point to help with experimental design for further functional elucidation of intracellular proteins/pathways/processes of interest. Given that certain drugs work on the modulation of intercellular protein interaction networks, the resource here may help with translational studies including drug development (e.g., probing the mechanisms of action of drugs and O-GlcNAcylation-targeting therapeutics). 

## Figures and Tables

**Table 1:** A list of 18 human OGT-interacting proteins with the highest identification events.

Entry Name (UniProt)	Protein Name	Gene Symbol	Number of Times Identified
HCFC1_HUMAN	Host cell factor 1	HCFC1	10
TET2_HUMAN	Methylcytosine dioxygenase TET2	TET2	9
BAP1_HUMAN	Ubiquitin carboxyl-terminal hydrolase BAP1	BAP1	9
OGA_HUMAN	Protein O-GlcNAcase	OGA	7
SET1A_HUMAN	Histone-lysine N-methyltransferase SETD1A	SETD1A	6
TRAK1_HUMAN	Trafficking kinesin-binding protein 1	TRAK1	6
OGT1_HUMAN	UDP-N-acetylglucosamine--peptide N-acetylglucosaminyltransferase 110 kDa subunit	OGT	6
TET1_HUMAN	Methylcytosine dioxygenase TET1	TET1	6
NUP62_HUMAN	Nuclear pore glycoprotein p62	NUP62	5
WDR5_HUMAN	WD repeat-containing protein 5	WDR5	5
N62CL_HUMAN	Nucleoporin-62 C-terminal-like protein	NUP62CL	4
SIN3A_HUMAN	Paired amphipathic helix protein Sin3a	SIN3A	4
DIDO1_HUMAN	Death-inducer obliterator 1	DIDO1	4
RBBP5_HUMAN	Retinoblastoma-binding protein 5	RBBP5	4
TAB1_HUMAN	TGF-beta-activated kinase 1 and MAP3K7-binding protein 1	TAB1	4
TRAK2_HUMAN	Trafficking kinesin-binding protein 2	TRAK2	4
ZEP1_HUMAN	Zinc finger protein 40	HIVEP1	4
TET3_HUMAN	Methylcytosine dioxygenase TET3	TET3	4

## Data Availability

The data presented in this study are available in the webserver OGT-PIN which is freely accessible (https://oglcnac.org/ogt-pin/; version: OGT-PIN_01.08).

## Supplementary Materials

Supplementary Materials are available online at https://www.mdpi.com/article/10.3390/ijms22179620/s1.
