# Supplementary material for: OGT Protein Interaction Network (OGT-PIN): A Curated Database of Experimentally Identified Interaction Proteins of OGT
Source: Int J Mol Sci. 2021 Sep 6;22(17):9620. doi: 10.3390/ijms22179620 (PMC8431785; doi:10.3390/ijms22179620)
Supplement: Supplementary file 1 [file ijms-22-09620-s001.zip › Supplementary Figures.pdf]

Supplementary Materials

OGT Protein Interaction Network (OGT-PIN): A Curated Database of Experimentally Identified Interaction Proteins of OGT

Junfeng Ma[1,*], Chunyan Hou[2], Yaoxiang Li[1], Shufu Chen[3], Ci Wu[1]

[1]Department of Oncology, Lombardi Comprehensive Cancer Center, Georgetown University Medical Center, Washington DC, USA.

[2]Dalian Institute of Chemical Physics, Chinese Academy of Sciences, Dalian, Liaoning, China.

[3]School of Engineering, Pennsylvania State University Behrend, Erie, Pennsylvania, USA

[*] Correspondence: junfeng.ma@georgetown.edu; Tel.: 1-202-6873802

Table of Contents

**Description**	**Page No.**
Supplementary Figure S1. A representation of the number of times that a specific interactor protein of OGT is identified.	S-2
Supplementary Figure S2: A word cloud representation of all human OGT interactors that appear frequently in OGT-PIN.	S-3
Supplementary Table S1: The list of all protein interactors of OGT and orthologues.	

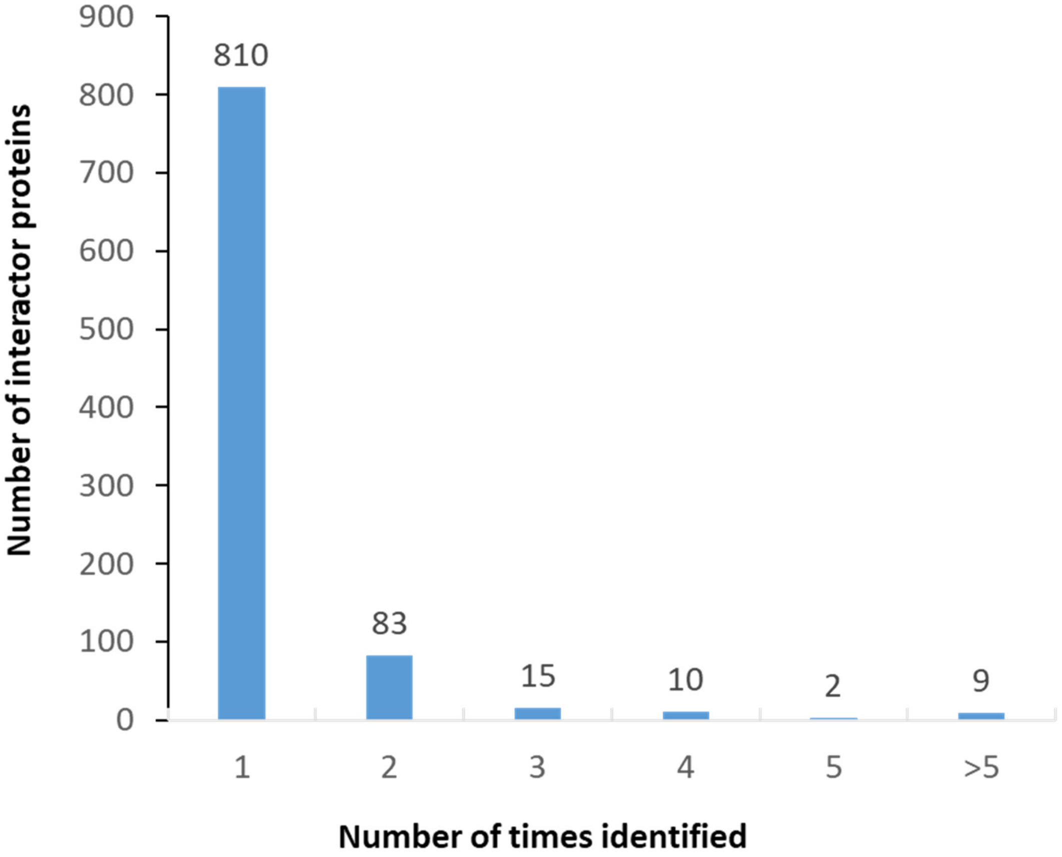

Supplementary Figure S1. A representation of the number of times that a specific interactor protein of OGT has been identified.

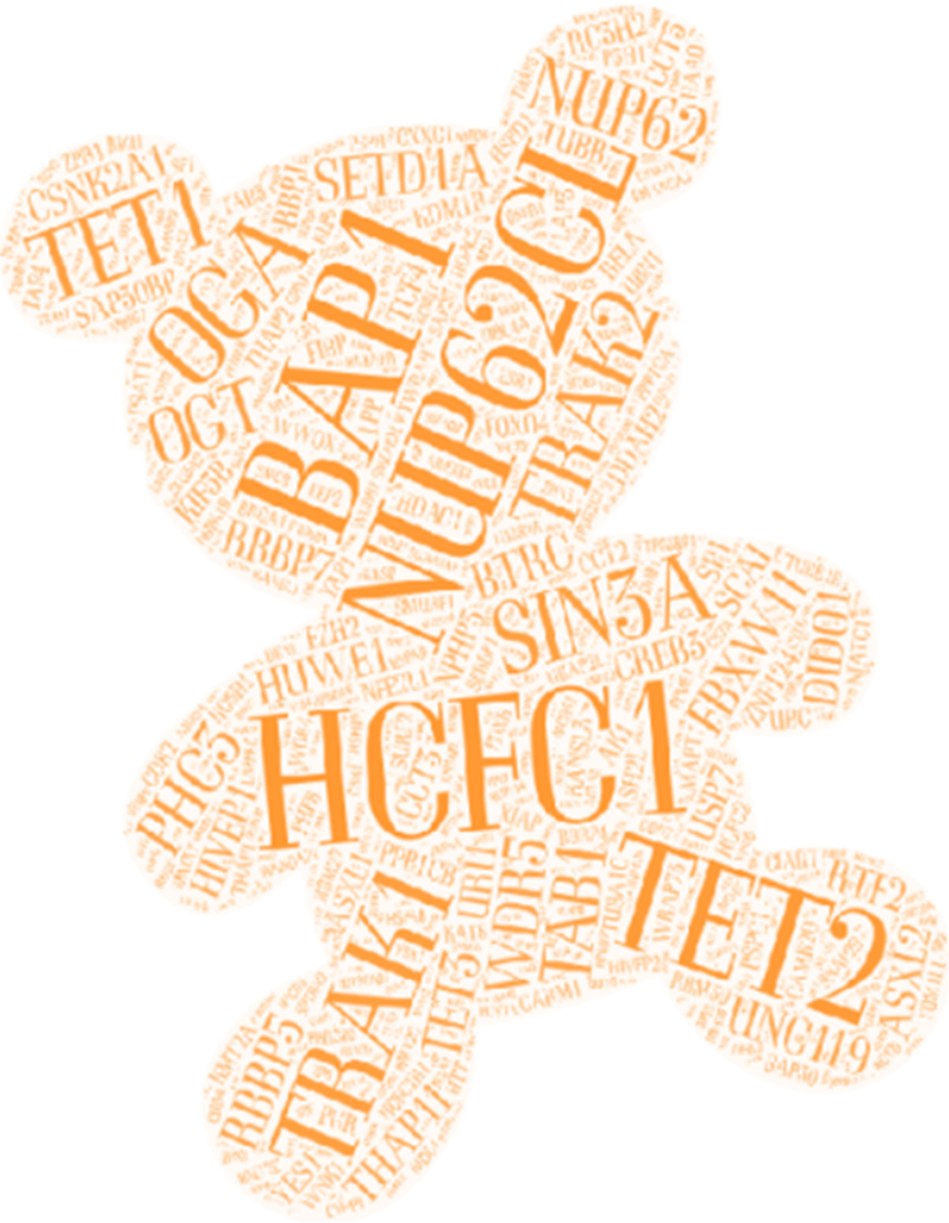

Supplementary Figure S2. A word cloud representation of 784 human OGT interactors that appear frequently in OGT-PIN. The size of each word indicates its frequency of overlap in the dataset.
